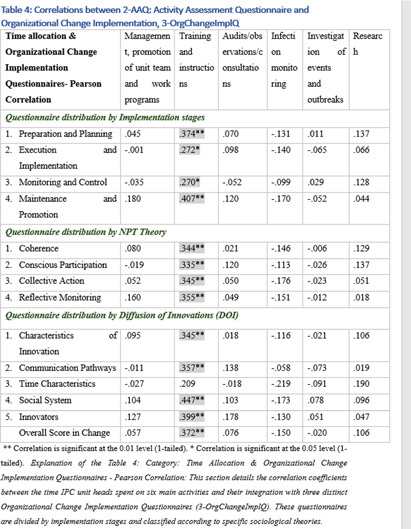# Managerial Influence on Infection Prevention and Control (IPC) Implementation in Israeli Hospitals: A Doctoral Research Study

**DOI:** 10.1017/ash.2024.250

**Published:** 2024-09-16

**Authors:** Dafna Chen, Stefan Cojocaru

**Affiliations:** National Center for Infection Control and Antibiotic Resistance, Tel-Aviv Sourasky Medical Center; Alexandru Ioan Cuza University from Iasi, Romania

## Abstract

**Background:** This research, part of a doctoral study, aims to examine the impact of managerial factors on the implementation of Infection Prevention and Control (IPC) measures in Israeli hospitals. The study focuses on identifying key facilitators and barriers from the perspectives of physician and nurse managers, with an emphasis on understanding the integration of managerial strategies and theoretical frameworks in IPC implementation. Objective: The objective is to explore specific managerial factors, both facilitators and barriers, influencing the effective implementation of IPC measures. The research investigates these influences through the lens of physicians and nurses managing IPC units in public hospital settings. Methodology: A mixed-method approach was adopted, involving in-depth interviews with ten IPC-Unit managers (five physicians and five nurses) and a comprehensive questionnaire distributed among IPC-Unit heads. The study’s demographic and professional profiles of participants are detailed in Table 1. The data collection process encompassed an Activity Assessment Questionnaire (2-AAQ) and an Organizational Change Implementation Questionnaire (3-OrgChangeImplQ), with the distribution of responses categorized by implementation stages and sociological theories (Tables 2-4). **Result:** Managerial autonomy emerged as a significant catalyst for IPC implementation, with supportive leadership and resource allocation being critical. Differences in approaches between physician and nurse managers were observed, reflecting diverse strategies in planning, execution, monitoring, and maintenance of IPC measures. The findings also revealed a natural alignment with sociological theories, particularly Normalization Process Theory (NPT) and Diffusion of Innovations (DOI), despite a lack of formal training in these areas. **Conclusions:** The study underscores the multifaceted nature of IPC implementation, highlighting the importance of managerial autonomy, supportive leadership, and a deep understanding of organizational culture. The inherent alignment of IPC strategies with NPT and DOI theories suggests the potential of these frameworks in guiding IPC implementation. The research advocates for the integration of these theoretical perspectives into formal training programs to enhance the effectiveness of IPC measures in healthcare settings.

**Figure:**
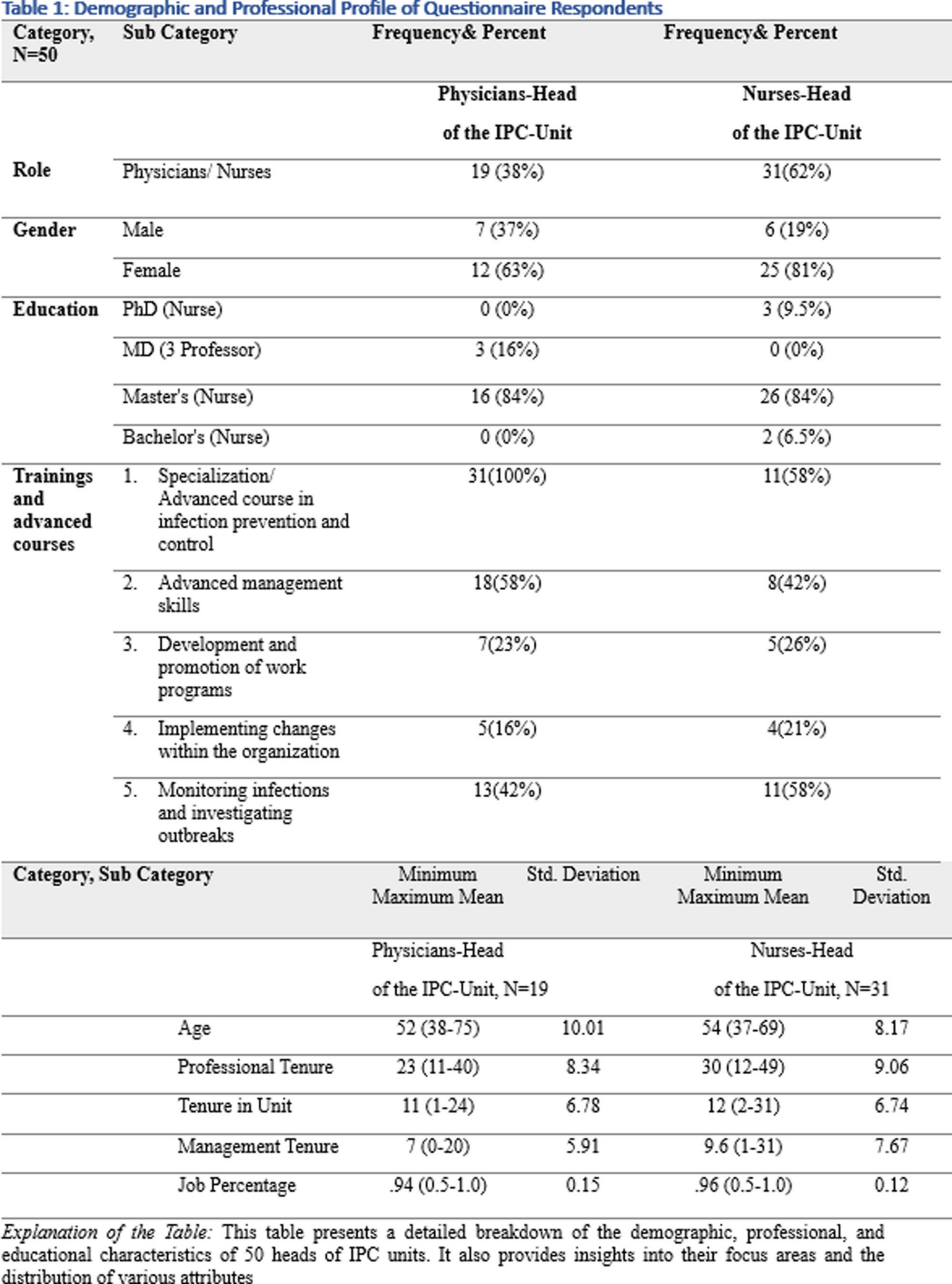

**Figure:**
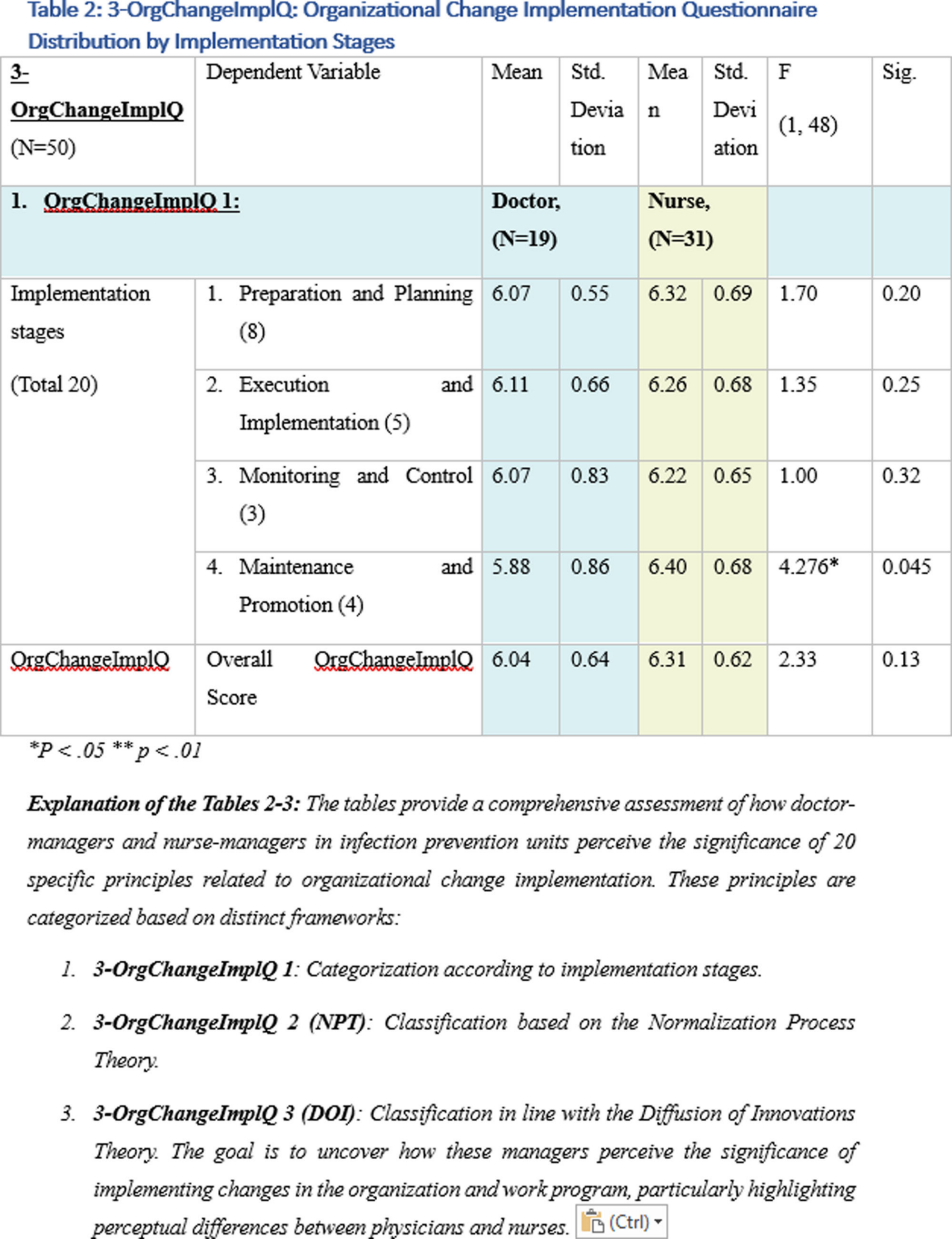

**Figure:**
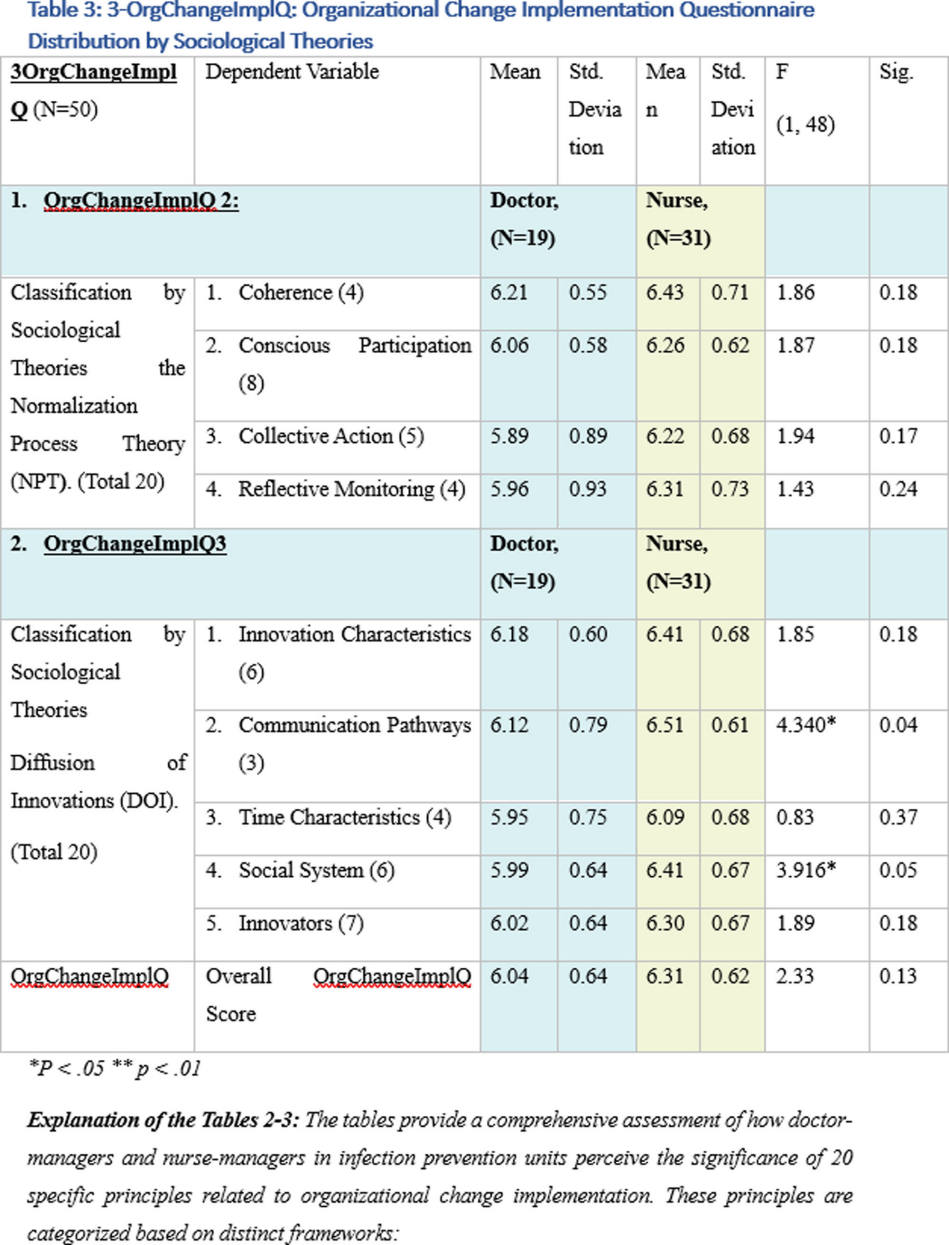

**Figure:**